# Managing NF2-associated vestibular schwannomas in children and young adults: review of an institutional series regarding effects of surgery and bevacizumab on growth rates, tumor volume, and hearing quality

**DOI:** 10.1007/s00381-020-04728-x

**Published:** 2020-06-16

**Authors:** Isabel Gugel, Julian Zipfel, Philip Hartjen, Lan Kluwe, Marcos Tatagiba, Victor-Felix Mautner, Martin Ulrich Schuhmann

**Affiliations:** 1 Department of Neurosurgery, University Hospital Tübingen, Tübingen, Germany; 2 Centre of Neurofibromatosis, Centre of Rare Disease, University Hospital Tübingen, Tübingen, Germany; 3 Division of Pediatric Neurosurgery, University Hospital Tübingen, Tübingen, Germany; 4 Department of Neurology, University Medical Center Hamburg-Eppendorf, Hamburg, Germany; 5 Department of Maxillofacial Surgery, University Medical Center Hamburg-Eppendorf, Hamburg, Germany

**Keywords:** Neurofibromatosis type 2, Vestibular schwannoma, Growth rate, Hearing preservation, Bevacizumab, Surgery

## Abstract

We reviewed our experience in managing of NF2-associated vestibular schwannoma (VS) in children and young adults regarding the effect of surgery and postoperative bevacizumab treatment. A total of 579 volumetric and hearing data sets were analyzed. The effect of surgery on tumor volume and growth rate was investigated in 46 tumors and on hearing function in 39 tumors. Long-term hearing follow-up behavior was compared with 20 non-operated ears in additional 15 patients. Sixteen operated VS were treated with bevacizumab. Mutation analysis of the NF2 gene was performed in 25 patients. Surgery significantly slowed down VS growth rate. Factors associated with a higher growth rate were increasing patient age, tumor volume, and constitutional truncating mutations. Immediately after surgery, functional hearing was maintained in 82% of ears. Deterioration of hearing was associated with initial hearing quality, larger tumor volumes, and larger resection amounts. Average hearing scores were initially better in the group of non-operated VS. Over time, hearing scores in both groups worsened with a similar dynamic. During bevacizumab treatment of residual tumors, four different patterns of growth were observed. Decompression of the internal auditory canal with various degrees of tumor resection decreases the postoperative tumor growth rates. Carefully tailored BAEP-guided surgery does not cause additional hearing deterioration. Secondary bevacizumab treatment showed heterogenous effects both regarding tumor size and hearing preservation. It seems that postoperative tumor residuals, that grow slower, behave differently to bevacizumab than reported for not-operated faster growing VS.

## Introduction

Neurofibromatosis type 2 (NF2) is an autosomal-dominant disorder caused by inactivation of the *NF2* gene on 22q12 resulting in the functional loss of its protein product merlin (moesin-ezrin-radixin-like protein) [1]. NF2 has a prevalence of 1:56.000 and an incidence of 1 case in 33,000 to 40,000 live births per year [2, 3]. Patients typically harbor tumors of the central nervous system (e.g., meningiomas, schwannomas, ependymomas), but schwannomas of the peripheral nervous system occur as well as a NF2-associated peripheral neuropathy [4]. The classical hallmark of NF2 is the occurrence of bilateral vestibular schwannomas (VSs), which, depending on their size, space occupying effect, and invasiveness are associated with progressive hearing loss, gait disturbances, vertigo, and facial palsy [4, 5].

Different to sporadic VS, NF2-associated VS (NF2-VS) are more likely to become symptomatic in children and young adults [6]. Known growth data from sporadic VS [7] cannot be transferred directly to NF2-VS because of a known higher proliferation index [8, 9], less vascularization, and a more lobular profile [10]. Truncating NF2 mutations (e.g., nonsense and frameshifting mutations) are known to result in more severe phenotypes, while non-truncating NF2 mutations (e.g., missense mutations) are rather associated with milder phenotypes [11–13]. Hearing-preserving treatment strategies are predominantly microsurgery [14] and chemotherapy, e.g., with the vascular endothelial growth factor (VEGF) inhibitor bevacizumab [15, 16]. The use of radiotherapy mostly in form of radiosurgery remains controversial in NF2 [17].

The strategies for hearing preserving microsurgery that have been applied partial resection after bony and dural decompression of the internal auditory canal with electrophysiological guidance by brainstem auditory evoked potentials (BAEP) and motor evoked potentials (MEP) of the facial nerve [18, 19]. Despite all care, surgery is always associated with a residual risk of damaging hearing due to unusual vulnerability of the cochlear nerve to minimal manipulations or vascular events like spasm of the labyrinthine artery elicited by tumor manipulation leading to a cochlear hypoperfusion [20]. Consequently, a conservative “wait and see” approach is often chosen, as for example in small and less aggressive tumors [21]. However, most young patients will need surgery sooner or later since there is progression over time. Furthermore, it seems conceivable that surgery for smaller tumors at a time of better hearing with existing BAEP guidance during surgery should have better chances of hearing preservation than surgery on large tumors at a time of already significantly impaired hearing without recordable intraoperative BAEP. Therefore, the timing of a surgical intervention is as difficult as “dosing” the procedure itself to balance the risks, benefits, and the patient opinion.

Anti-VEGF treatment using, e.g., bevacizumab of NF2-VS has been used in adults to stabilize tumor growth to sustain or improve hearing function, but mostly it was applied to non-operated larger tumors [16, 22, 23]. Since the long-term side effects of VEGF inhibitors are of concern when the use is already started in adolescence, the application of, e.g., bevacizumab in this age group remains controversial. It seems desirable to postpone such therapy as long as possible; thus, it is used in our institution as second-line approach after surgery. It is unclear, however, if application at a time point after surgery will have comparable effects as previously published in “untouched” NF2-VS [16].

This review paper summarizes our institutional experience with adolescents and young adults with NF2 regarding the VS management, aiming at preservation of a functional hearing as long as possible. We related to and condense the results of three original publications on the topics of growth rate [24], effects of surgery on hearing [25], and effects of second line bevacizumab [26] into one review, to create a comprehensive overview on the topic for the reader. We discuss the results in the light of the recent literature. For further details, the original publications should be studied.

## Methods

We retrospectively reviewed NF2 patients from 2004 to 2018 who were monitored or treated for NF2-VS at the Centre of Neurofibromatosis Tübingen. The diagnosis was confirmed by clinical evaluation using the Baser diagnostic criteria for NF2 [5]. These retrospective studies were approved by the ethics board of the Medical Faculty and University Hospital of Tübingen. We selected only NF2 patients younger than 25 years at the time of diagnosis with bilateral VSs, availability of a minimum of 2 preoperative and postoperative MRI scans of sufficient extend and image quality, and a minimum follow-up of 12 months. Overall, 28 patients met the inclusion criteria. Fifteen patients with a less aggressive course of disease did not require surgical intervention for the NF2-VS and served as a control group.

In the analysis of growth rates before and after surgery in relation also to clinical factors, 28 patients were included. Eighteen patients received bilateral and 10 unilateral surgery, and two tumors were operated on twice, resulting in 48 interventions on 46 tumors in 28 patients. For volumetric analysis, postcontrast thin-slice (≤ 3 mm) T1-weighted MR images were imported into iPlan Net software (Brainlab) and the volume of the VS on each side was determined using manual segmentation by one investigator (IG). In total, we analyzed 579 MRI data sets. Growth rate was calculated using at least 2 MRI data sets prior to and after surgery. After surgery, the growth rate determination was restarted from the first MRI performed usually 3 months after surgery [24].

To estimate the tumor growth rate, a robust multilinear regression model was fitted on a MatLab 2016 Engine (MathWorks). The algorithm uses iteratively reweighted least squares with a bi-square weighting function. The robust fit is less influenced by outliers. It returns coefficient estimates for a robust multilinear regression of the tumor volumes measured at the different time points [27]. Pearson’s correlation coefficient was used to analyze the relationship between VS volume, age at the time of diagnosis, and VS growth rate using the Statistical Package for the Social Studies (SPSS 10.0 for Windows, SPSS Inc.). ANOVA with the Games-Howell post hoc test was performed to compare the growth rate in 3 age groups (group 1, ≥ 10–< 15 years; group 2, ≥ 15–< 20 years; and group 3, ≥ 20–< 25 years). A stepwise multiple regression was applied to predict the influence of sex, family history, mutation type, overall tumor load (presence of intracranial, spinal, and peripheral tumors), and tumor side and size categorized by the Hannover Classification System [28] on preoperative and postoperative growth rates.

For the analysis of the effect of surgery on hearing quality, 23 patients with full hearing data sets were included with surgery performed on 39 tumors. Patients with hearing aids or implants (e.g., cochlear or auditory brainstem implant) were excluded. Twenty control ears in 15 NF2 patients with the same cut-off age at time of diagnosis served as a control group. These patients belonged to a cohort with different tumor behaviors, had stable and good hearing with little tumor growth, and therefore did not require a surgical intervention during the observation period. Neither the operated nor the non-operated patients were treated with bevacizumab

Indications for surgery were (a) large tumors (T4 Hannover Classification [28]) on both sides with brainstem compression or (b) continuing tumor growth and deterioration of BAEP and/or impairment of pure-tone average (PTA) and/or speech discrimination score (SDS) during observation. All surgical interventions were performed via a retrosigmoid approach with primary bony and dural decompression of the internal auditory canal (IAC), followed by various resection amounts under continuous neurophysiological monitoring by two experienced neurosurgeons (MT, MUS).

Regarding the impact of surgery on hearing outcome, we stratified for age at diagnosis, preoperative tumor growth rate, preoperative tumor volume, resection volume, and type of constitutional mutation. Mutation analysis data was available for 21 of 23 patients.

Hearing was assessed in all patients by regular 4-frequency pure tone average (PTA), speech discrimination score (SDS), and brain stem auditory evoked potential (BAEP) within 4 weeks before surgery and every 3 to 6 months thereafter. Hearing data were classified using the Gardner and Robertson Scale (G-R) [29], American Association of Otolaryngology–Head and Neck Surgery Scale (AAO-HNS) [30], and BAEP Classification System [19], direct and mean values of PTA, SDS, and BAEP waves including interpeak latencies (IPL). Preservation of hearing was defined as PTA and SDS change of ≤ 15 dB or ≤ 15% compared with the preoperative value [31] and by maintenance of G-R, AAO-HNS, and BAEP class. Facial nerve function was categorized by the House–Brackmann Classification System (H–B) [32] before surgery and 3 months after surgery.

Hearing before and after surgery was compared using paired by *t* test with two-tailed hypothesis. Pearson´s correlation coefficient was used to analyze the relationship between hearing values/classifications and VS growth rate, volume, and resection amount. After normalization of preoperative and postoperative hearing data, an analysis of variance (ANOVA) with the Games–Howell post hoc test was carried out with PTA and SDS values compared with age of patients. To investigate the long-term course of PTA and SDS values before (24 months) and after (until 42 months) surgery, an analysis of covariance (ANCOVA) was performed and data compared with the 20 control ears.

To investigate the effect of second-line bevacizumab on tumor growth and hearing function, 9 patients harboring 16 NF2-VS who had undergone previous surgery were analyzed. Indications for bevacizumab treatment after surgery were continued tumor growth and/or further hearing deterioration of BAEP and/or impairment of PTA and/or SDS. For 8 of the 9 patients, mutation analysis was available. Adverse events and toxicity data were collected during follow-up visits and scored according to the National Cancer Institute’s Common Terminology Criteria for Adverse Events, version 5.0. In the treatment periods, patients received intravenous bevacizumab 5 mg/kg every 2 weeks. In cases with stabilized tumor growth and hearing, the dose was gradually reduced to minimize long-term toxicity effects.

Tumor volumetry and determination of growth rate and resection amount were performed, classified, and calculated in 284 data sets as described above. Hearing was assessed in all patients as described above. Hearing deterioration was calculated as annual percentage deterioration compared with baseline value. Tumor volume, PTA, and SDS data were separated into 3 groups: (1) before surgery, (2) after surgery in the non-treatment periods, and (3) after surgery and in the bevacizumab-treatment periods. Change of tumor volume, PTA, and SDS between two measurement points was calculated as change/months. Since tumor size varies largely, the percentage change of tumor volume between two measurement points was normalized by dividing it with the tumor volume of the first one as follows: 100 × [volume 2 − volume 1]/[measurement 2 – measurement 1] / [volume 1]. Values in the treatment and the non-treatment periods were compared using a *t* test with two-tailed hypothesis and presumed equal variation. Furthermore, a multiple regression analysis tested the influences of preoperative and postoperative tumor volume, postoperative relative growth rate, and resection amount on the relative tumor growth rate under bevacizumab treatment.

## Results

### Effect/influence of surgery and clinical factors on tumor growth rate and volume

Regarding the relation of growth rate to clinical factors and surgery, the included 28 patients had a mean age at diagnosis of 12.75 ± 5.80 years (range 1–22 years) and at the first surgery of 15.39 ± 4.65 years (range 8–26 years), and that at the second surgery was 16.39 ± 4.23 years (range 11–24 years). The mean overall follow-up before surgery was 20.51 ± 17.74 months (range 13–63 months) and after surgery 75.36 months (range 21–167 months). The tumor volumes at the time of diagnosis ranged from 0.01 to 11.04 cm^3^ (mean 1.52 ± 2.49 cm^3^) and the preoperative growth rates from 0.04 to 3.75 cm^3^/year (year) (mean 0.60 ± 0.82 cm^3^/year).

There was a significant correlation of tumor volume at the time of diagnosis with age at diagnosis (*r* = 0.359, *p* = 0.009) and with tumor growth rate at time of diagnosis (*r* = 0.407, *p* = 0.006). When comparing growth rates in the 3 age groups, preoperative growth rates of VS were significantly higher in older patients.

In the multiple regression model, a significant influence was only seen between tumor size and preoperative growth rate (*p* = 0.001, *B* = 0.79, SEB = 0.21, *β* = 0.56) as well as mutation type and postoperative growth rate (*p* = 0.018, *B* = 0.31, SEB = 0.12, *β* = 0.16) (*B* = unstandardized regression coefficient; SEB = standard error of the coefficient; and *β* = standardized coefficient). All other factors had no significant influence on preoperative or postoperative growth rates.

*NF2* mutations were found in 18 (72%) of the 25 patients who had genetic results from peripheral blood. Eleven of 18 had truncating mutations such as nonsense and frameshifting mutations. 6/18 had splicing mutations, and 1 had a large genome alteration. There was no missense mutation. Four patients were defined to be NF2 mosaic. No mutation could be detected in 3 patients. A significantly higher postoperative growth rate was found for NF2-VSs of patients with constitutional truncating mutations (0.40 ± 0.49 cm^3^/year) compared with NF2-VS with constitutional splicing mutations (0.04 ± 0.27 cm^3^/year). Total resection was accomplished in 1 tumor, near-total resection in 3, and subtotal resection in 2 tumors. Partial resection of different degrees was achieved in the vast majority of 39 tumors, and finally bony decompression only was done in 3 patients. The single patient with gross-total resection experienced no recurrence and thus had no postoperative growth rate calculation.

Surgery reduced the tumor volume on the first postoperative scans in all cases but one (with bony decompression only). Pooling preoperative and postoperative data from 33 tumors, we found a mean of 3.34 ± 4.97 cm^3^ tumor volume before surgery and a highly significant reduction by approximately 50% to 1.66 ± 3.82 cm^3^ after surgery (*p* < 0.001).

In 31 NF2-VS, growth rates before and after surgery could be compared. In 3 tumors, the growth rates did not change after surgery. The mean growth rate of all tumors was 0.69 ± 1.30 cm^3^/year before surgery, that was reduced by approximately 33% to a postoperative growth rate of 0.23 ± 0.42 cm^3^/year (*p* = 0.013).

Despite the fact that small (T1 and T2) NF2-VS had a significantly (*p* = 0.001) lower preoperative growth rate of 0.24 ± 0.45 cm^3^/year than large (T3 and T4) tumors with 1.21 ± 1.78 cm^3^/year, no such difference was seen in the growth rates after surgery.

### Effect/influence of surgery on hearing quality

The 32 patients included in this analysis had a mean age at diagnosis of 12 ± 7 years (range 1–22 years), a mean age at first surgery of 16 ± 5 years (range 8–26 years), and at second surgery of 16 ± 4 years (range 11–24 years). Mean overall follow-up was 75 ± 6 months, range 21–167 months. 32/39 investigated tumors underwent a partial resection. There was a statistically significant difference (*p* = 0.002) in preoperative tumor volume (2 ± 2.6 cm^3^, range 0.1–10.5 cm^3^) to postoperative tumor volume (1 ± 1.6 cm^3^, range 0–18.6 cm^3^). Mean growth rate after surgery (0.3 ± 0.4 cm^3^/year, range − 0.01–2.2) was approximately 50% of preoperative growth rate (0.6 ± 0.7 cm^3^/year, 0.03–3.4 cm^3^/year, *p* = 0.03).

There were no major surgical complications in the short- and long-term follow-up (e.g., intraoperative bleeding, intraoperative air embolism, cerebrospinal fluid fistula, infection, neurologic deficit) other than a deterioration of hearing (see below). Facial nerve function was unchanged in 37/39 (95%) tumors. In two cases, an improvement from H-B grades II to I occurred after decompressive surgery. In 8 cases, minor complications directly after surgery such as wound healing disorders (2/8) and severe neck pain (6/8) occurred and were treated conservatively.

#### Short-term quality of hearing after surgery

In 32/39 ears (82%), a *functional hearing* was preserved after surgery. There was a slight but significant (*p* = 0.009) reduction in pure tone average (PTA) from preoperatively 17 ± 16 dB (range 1.3–80 dB) to 21 ± 18 dB (range 1.3–78 dB) postoperatively. A similar slight drop was found for the mean speech discrimination score SDS from 85 ± 27% (range 0–100) to 81 ± 32% (range 0–100). All those changes were functionally not relevant. One ear improved in SDS though not in PTA.

A preservation of the *preoperative hearing class* could be achieved for 29 (74%), 28 (72%), and 24 (62%) ears regarding G-R, AAO-HNS, and BAEP, respectively. Three ears (8%) improved in BAEP. A better preoperative G-R grade correlated with a higher preservation rate in the same grades: 81%, 50%, and 33% for grades I, II, and III, respectively. Further correlation parameters to postoperative hearing are demonstrated in Table 1.

**Table 1:** Correlations of parameters with postoperative hearing. This data was published in Gugel et al. [25]

	Correlation
Positive correlation between -Preoperative BAEP and postoperative PTA -Preoperative PTA and postoperative PTA -Preoperative SDS and postoperative SDS	*r* = 0.3, *p* = 0.04 *p* < 0.001 *p* < 0.001
Negative or inversed correlation between -Truncation NF2 mutations and worse PTA (compared with splicing mutation) -Truncation NF2 mutations and worse SDS (compared with splicing mutation) -Larger preoperative tumor volume and worse postoperative PTA -Larger resection amount and worse postoperative PTA -Larger resection amount and worse postoperative SDS	*p* = 0.012 *p* = 0.008 *r* = 0.3, *p* = 0.04 *r* = 0.354, *p* = 0.031 *r* = − 0.386, *p* = 0.018

*BEAP*, brainstem auditory evoked potential; *PTA*, pure-tone average; *SDS*, speech discrimination score; *NF2*, neurofibromatosis type 2

In the remaining 7 of 39 ears, surgery resulted in *postoperative deafness* or *loss of functional hearing*. 2/7 ears had a non-functional hearing already before surgery. In the remaining 5 ears, preoperative BAEP were already severely affected. Of note is that in these 5 ears, the preoperative tumor volumes and obtained resection amounts were significantly larger compared with the 32 ears with preserved functional hearing: tumor volume 3.7 ± 3.8 cm^3^ vs. 1.6 ± 2.0 cm^3^ (*p* = 0.027), resection amount 66 ± 38 % vs. 38 ± 32 % (*p* = 0.024), respectively.

#### Long-term quality of hearing after surgery

The postoperative follow-up of 32 ears with functional hearing regarding PTA and SDS was up to 42 months. The comparison with 20 age-matched ears in 15 patients of the control group (age at diagnosis 10 ± 7 years, range 0–21 years), which did not receive surgery, showed—as expected—that the initial hearing of the non-operated control group was better, because this unimpaired hearing was the reason for not receiving surgery. However, the mean values of hearing scores in both groups worsened gradually (*p* < 0.05) in a similar dynamic over the 42-month (postoperative) follow-up period. No accelerated hearing deterioration was evident for the operated cases despite a worse hearing starting point. Rather, the gap between the decreasing lines of the two groups tended to become smaller at the end of the follow-up period.

### Effect of second-line bevacizumab treatment after previous surgery on tumor growth rate, volume, and hearing

The 9 patients with 16 ears included in this analysis had a mean age at diagnosis of 12 ± 7 years (range 1–20 years), an age at surgery of 16 ± 5 years (range 8–23 years), and an age at beginning of bevacizumab treatment of 19 ± 4 years (range 14–26 years). The mean preoperative tumor volume was 2.3 ± 2.8 cm^3^ (range 0.2–10.5 cm^3^), which was reduced by surgery to a postoperative mean volume of 0.9 ± 1.4 cm^3^ (range 0.03–5 cm^3^). At the time bevacizumab treatment was initiated, the tumor volume had increased again to a mean of 2.0 ± 1.8 cm^3^ (range 0.2–6.5 cm^3^). The mean postoperative follow-up period *without* bevacizumab (BVZ) was 36 ± 26 months (range 13–63 months), and *with* BVZ, it was 28 ± 14 months, (range 7–43 months).

In 2 patients, no adverse effects of bevacizumab were observed in the entire treatment period. Five patients had mild adverse effect like fatigue and/or dryness of skin and mucous membranes. Two patients experienced proteinuria and arterial hypertension (grade III adverse events) requiring medication with angiotensin-converting enzyme (ACE) inhibitors and one received a beta blocker in addition during the first 6 months. Bevacizumab treatment had to be transiently discontinued but could be resumed after 3 months. Antihypertensive medication could be limited in one to the short treatment-free interval and in the other, it was continued until the end of observation period in reduced dosage.

In 7 of the 9 patients, data were available for bilateral NF2-VS. In each of these patients, the response patterns were comparable on both sides, and in 5 patients, the growth curves were basically parallel to each other.

The growth during the treatment and the non-treatment periods clustered into 4 patterns.

- Pattern 1 (*n* = 1): growth in the 1st non-treatment period stopped/decreased in the following 2 treatment periods.
- Pattern 2 (*n* = 2): growth in 1st treatment period and suppressed growth in the 2nd treatment period.
- Pattern 3 (*n* = 2): continued growth in both the treatment and the non-treatment periods.
- Pattern 4 (*n* = 4): no or low growth in both the treatment and the non-treatment periods

**Fig. 1:**
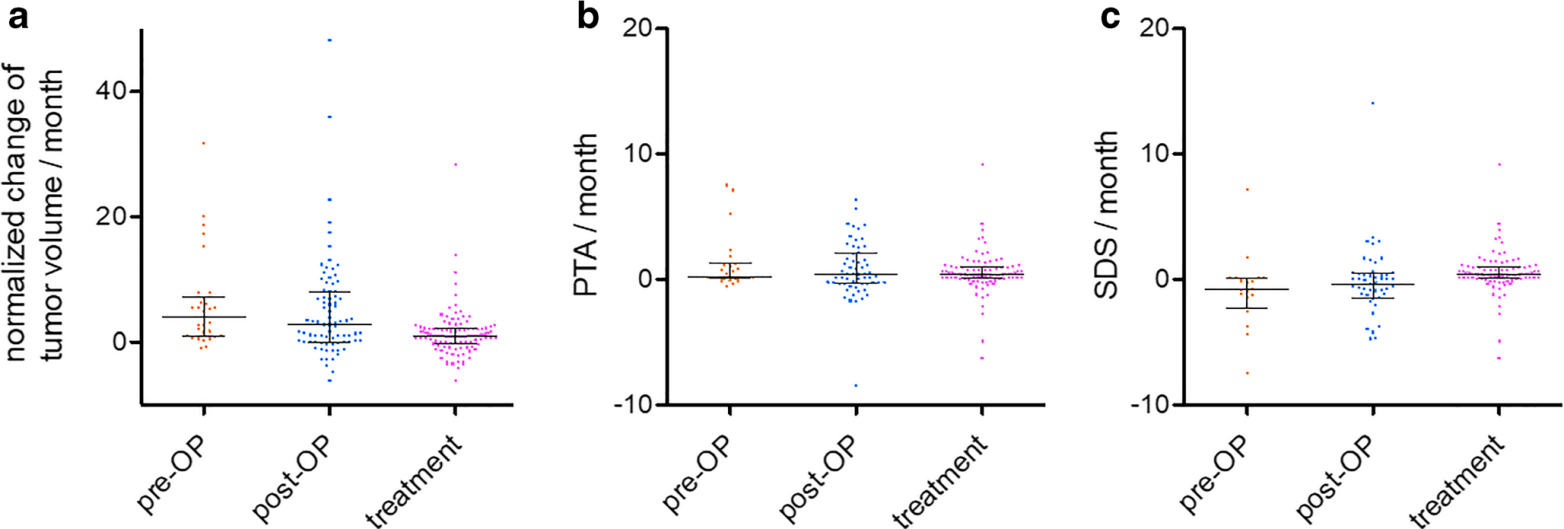
Box-plots showing change of tumor volume (**a**), changes of pure-tone average (PTA) (**b**), and of speech discrimination score (SDS) (**c**) between two measurement points in the treatment and the non-treatment periods, as well as in the period before surgery. For each parameter, the data sets in the three periods did not differ significantly from each other but a trend of slower growth and change of SDS in treatment periods was seen. Preop, preoperative; Postop, postoperative. This data was illustrated and published in the original work of Gugel et al. [26]

Growth between two measurement points was slightly lower in the treatment period than in the non-treatment period. However, this difference was not significant (*p* = 0.19) and the variations were large (Fig. 1a).

The effect of treatment on hearing was very heterogenous. Rapid hearing deterioration in the non-treatment periods was observed in 4 patients. However, rapid or continued hearing deterioration was also observed in treatment periods of 4 patients. In some cases, hearing deterioration correlated with tumor growth; however, in others cases, it did not.

Consequently, the overall PTA increases between two measurement points were comparable in the treatment and the non-treatment periods (Fig. 1b). The SDS decreases between two measurement points were slightly less in the treatment periods than in the non-treatment periods (Fig. 1c), and the difference however was not significant (*p* = 0.13). The variations were large for both the PTA and the SDS data.

## Discussion

The purpose of this review of an institutional series of children, adolescents, and young adults < 25 years at the time of diagnosis of NF2 is to summarize and condense our findings regarding:

A. preoperative and postoperative growth rates and the influencing factors on growth of NF2-VS
B. the effect of a surgical strategy aiming at hearing preservation on the resulting preservation rate of functional hearing and on the long-term effect of surgery on hearing preservation
C. the effect of second-line bevacizumab treatment on previously surgical treated NF2-VS.

The extensive analysis with in depth information on all details and subgroups including supplementary information can be found in the 3 original publications [24–26].

The literature on VS management for this specific age group of NF2 patients, who are often treated in adult neurosurgery centers, which are not necessarily NF2 centers, is sparse. The natural course of growth rates in those patients that manifest early, thus, often belongs to the clinically more severely affected Wishart phenotype, and the effect of surgery on growth rates is not well described and is hampered by a heterogenous course of the disease in the individual patients and the heterogenous ways of assessing growth rate in literature so far.

The effect of second-line bevacizumab (BVZ) on previously surgically treated NF2-VS at a time of secondary hearing deterioration has as well not been published, since experiences were mostly gathered from previously untreated NF2-VS. Although we report only on a small series of patients and results have thus a preliminary character, the first impressions of a different effect of BVZ in this group of patients than reported for untreated patients seem important.

### Results of growth rates

Our analyses differ from a technical point to previously reported series. First, the majority of studies used diameter-based methods and 2-box models [1, 33–41] to estimate tumor growth rates but not automated/semiautomated 3D MRI volumetry. Harris et al. showed in NF2-associated VSs that linear measurements underestimated VS growth rate compared with volumetric measures by semiautomated segmentation software [42]. Although more laborious and certainly time consuming, volumetric measurements are clearly more sensitive and precise and should become, as demonstrated herein, the gold standard for VS monitoring in NF2 patients instead of linear measurements [42, 43].

Regarding MRI scan quality, the thickness of MRI slices is crucial. In our study, volumetry was carried out only on MRI scans with a slice thickness < 3 mm. In many previous studies, thickness of MRI slices varied largely and/or details for these parameters are not given. For instance, Slattery et al. used MRI scans of varying thickness and qualities for a diameter-based growth rate calculation. In their linear dimension, a change had to be larger than 5 mm to reach a statistical significance [41]. Third, only 4 studies focused on young NF2 patients with a compatible age range [33, 34, 38, 44] and among them, only one study used volumetry-based calculation for VS growth rate [44].

Previous studies reported a controversial correlation between patient age and VS growth rate, some even an inversed correlation [33, 36, 38, 39]. ^.^ Only one study reported an increasing growth rate with increasing age [1] as in our cohort. By defining growth rate as the annual *volume* changes, preoperative growth rates correlated with the age of the patient. This matches with the finding that preoperative tumor growth rate and volumes correlate with each other, and volumes were smaller in earlier (younger) diagnosed patients than in older ones. Consequently, children with smaller tumors had lower growth rates than teens and young adults with larger tumors which had higher growth rates per year. Likewise, Li et al. showed that growth rates of VSs were generally lower at younger age at symptom onset and correlated with initial VS volume [37].

One main finding was that—contrary to our expectation that surgery would not affect growth rate (null hypothesis)—we actually saw that in all but 3 tumors, a significant postoperative reduction in growth rate occurred. Possible explanations are the pure effect of tumor volume reduction, since we demonstrated that, preoperatively, smaller tumors grew at a slower rate. Secondly, it has been shown in a pilocytic astrocytoma cell model that mediators of inflammation such as interleukin-1b (IL-1b) did reduce cell growth via induction of senescence-associated secretory phenotype expression [45]. It is well known from pilocytic astrocytoma surgery that residual tumors often switch into senescence after resection despite significant growth prior to surgery. Since surgery induces a postoperative inflammatory reaction, a similar mechanism might occur after partial resection of VS.

Patients were rather young at onset and had multiple additional tumors such as spinal ependymomas, intracranial meningiomas, and non-vestibular schwannomas. Not surprisingly, many had a high tumor load. In this setting, we could demonstrate for the first time in literature that patients with truncating mutations had higher postoperative (not preoperative) growth rates than those with splice-site mutations. Genotype-phenotype correlations and their consequences on severity of NF2 disease in general have been well investigated [46]. Truncating mutations, like nonsense or frameshift mutations, are associated with a younger age at onset, a higher tumor burden, and a more severe course of disease than missense or splice-site mutations [11, 46]. So far, no context of VS growth rate and mutation type could be seen in NF2 patients. Irving et al. investigated the influence of mutation type on a clinical growth and tumor cell proliferative index in NF2-associated VSs (but did not calculate growth rates) and detected no correlation [47].

Keeping in mind that a positive correlation of mutation type and postoperative tumor growth rate in VSs was so far not described, we cannot exclude that an additional causal factors in tumor microenvironment (e.g., growth factors), which can be activated or released due to surgical manipulation, did influence higher postoperative growth rate in patients with truncating mutations. This phenomenon has been described in residual primary breast cancer with an increase of VEGF and pro-MMP-9 produced in the surgical field [48].

Based on our preliminary finding, we suggest that patients carrying truncating mutations should be monitored more closely, e.g., every 3 months, after surgery than patients with other types of mutations.

#### Results of strictly hearing preserving surgical strategy

Due to the high risk of bilateral deafness over time, hearing preserving surgery has a much higher value for younger than older NF2 patients and in general is of higher importance than for patients with unilateral sporadic VS. Early experiences in NF2 patients of all age groups could already demonstrate that total resection has a much lower hearing preservation rate of 30%, compared with 73% achieved by subtotal resection [18]. We report a very high postoperative functional hearing preservation rate of 82% after BAEP-guided surgery in NF2 patients, operated—if seen early enough—at the time of beginning of, otherwise at time of significant hearing deterioration. Operating at the ideal time point seems crucial. This study confirms previous findings that hearing preservation is more likely to be achieved in patients that have a good hearing before the surgery [18, 49, 50]. Our results including the preoperative BAEP data confirmed this rule and suggest that intervention should be considered when BAEP starts to deteriorate, before a drop in PTA or SDS can be detected.

The study also underscores that the surgeon needs to be conscious with regard to the intraoperative BAEP behavior and, in general, needs to show a defensive surgical behavior to preserve functional hearing. Those patients who lost hearing in our series had, apart from larger preoperative tumor volumes and worse hearings, also larger tumor amounts removed. Strict compliance to significant changes in BEAP will result in smaller resection amounts in the majority of cases. We generally react to any decrease of BEAP and wait until recovery before continuing tumor reduction. A deterioration of more than 50% of BEAP amplitude with a recovery time of more than 2 min certainly is a stop sign that should result in discontinuation of tumor volume reduction. A similar experience of better hearing preservation by deliberate partial resection has been reported by Wigand et al. [51] many years ago in non-NF2 patients.

The second major finding of our investigation was that long-term course (mean 6.28 years) of gradual hearing deterioration became similar in cases which needed initial surgery, thus, had more aggressive tumors leading to hearing deterioration, and those without surgery, since their tumor grew slower from the very beginning and hearing remained more stable. The variation of hearing deterioration was large but equal in both groups of tumors, regardless of surgery. This observation suggests that surgery of NF2-VS did not accelerate hearing loss but one the contrary modified the initial, more rapid hearing deterioration toward a slower hearing deterioration, which was similar to that of the non-operated less aggressive NF2-VS. This can most likely be explained by the reduction in growth rate that occurred after surgery.

#### Effect of second-line bevacizumab after previous surgical intervention

So far, the published experience on bevacizumab treatment for vestibular schwannomas in NF2 patients describes mainly progressive and large tumors without previous surgery and positive/promising results for both suppressing radiological tumor growth and for preserving/improving quality of hearing have been reported [16, 23, 52]. By contrast, our study included rather small residual tumors after partial resection with a postoperatively reduced growth rate. This difference may be one main reason for the discrepancy in efficacy of bevacizumab treatment in this series, where we found very heterogenous effects. In some patients, tumor growth rates slowed down, others responded partially and others not at all. In the group comparison, growth rates were decreased under bevacizumab but the significance level was not reached due to the high data scattering in a small series of 16 tumors in 9 individuals. Furthermore, bevacizumab treatment was started at a delayed time point compared with a primary treatment and thus the overall hearing impairment was more advanced at time of treatment start. In none of the cases, we in addition observed a radiological regression of the tumor or improvement of hearing. Therefore, the expectation to a second-line bevacizumab treatment has to be different; it is used to further slow down growth and loss of hearing and according to this first analysis, we counsel our patients in this regard and cannot promise tumor volume reduction and hearing improvement, as we have observed—as described in literature—in those individual off-label treatments we have conducted on larger non-operated NF2-VS.

Despite a known genotype-phenotype correlations in NF2 patients [53, 54], its influence on the response of bevacizumab has not been investigated in detail. Our preliminary data suggest that patients with unsatisfactory or mixed response to bevacizumab (pattern 2 and 3) have a more severe genotype and phenotype (truncating mutations and high tumor loads). However, the number of patients is too low to draw conclusions at this time point. In addition, the intensity of VEGF-/VEGFR expression in the tumor material should be taken into account, since a correlation to tumor growth or effect of therapy has been described [55, 56]. Therefore, targeted studies to determine a possible medication response and dosage are necessary in times of individualized medicine.

The last but important concern for using bevacizumab in adolescents and very young adults is the unknown effects of prolonged treatment, since it is known that particularly dermatological, cardiovascular, and renal side effects will occur in up to 50% of the patients [23]. As described herein, the phenomenon of accelerated rebound growth and concomitant hearing deterioration in drug holidays, which were necessitated by adverse events, should not be underestimated. Therefore, a close monitoring of blood pressure and proteinuria and rather early antihypertensive treatment with, e.g., ACE inhibitors is mandatory, to counterbalance side effects early and avoid a complete cessation of treatment.

## Conclusion

Children and young adults with NF2 are extremely dependent on hearing preservation for as long as possible to enable their continuation of social development and education. Under consideration of functional preservation of hearing, early diagnosis of the disease and monitoring of these tumors and their growth rate by 3D tumor volumetry seems very important to be informed of changes “online” and not taken by surprise. Hearing-preserving treatment strategies are crucial and differ significantly from the strategy “remove tumor and preserve facial nerve function” as often applied in non-NF2 patients. If transferred to adolescents and young adults with NF2-VS, this approach “steals” life time of good hearing quality, as we have often seen in patients we saw later in their life. The identification of risk factors for faster tumor growth has to be considered helpful for treatment planning to determine the ideal time point of surgery.

The surgical strategy we developed aims strictly at hearing preservation and not at the amount of tumor volume reduction or total resection. The effect of a consequent application of a “hearing first” strategy on quality of hearing has not been previously reported for this age group where it seems to matter most. The results are promising and we advocate this approach as first-line treatment, to delay the onset of bevacizumab treatment, which invariably will be, if effective, a long-term treatment with considerable side effects in the majority of patients.

It seems, however, that the effect of BVZ is less prominent after primary surgical intervention due to smaller tumors with slower growth rate and an already impaired hearing, and patients have to know that this is another means of prolonging time with useful hearing for mostly years, before strategies for hearing restoration have to be applied. Those options are described by in this special issue.

## Data Availability

This work is a summary of three original articles which has been published before (Gugel et al. Cancers (Basel). 2019 Nov 25 [26]; Gugel et al. J Neurosurg Pediatr. 2019 Aug 23 [24]; Cancers (Basel). 2019 Sep 16 [25]). Detailed data and original illustrations and figures are given in these original works.
